# Supplementary material for: Read Operation Mechanism of Feedback Field-Effect Transistors with Quasi-Nonvolatile Memory States
Source: Nanomaterials (Basel). 2024 Jan 18;14(2):210. doi: 10.3390/nano14020210 (PMC10819914; doi:10.3390/nano14020210)
Supplement: Supplementary file 1 [file nanomaterials-14-00210-s001.zip › nanomaterials-2818847-supplementary.pdf]

Supplementary information

Read Operation Mechanism of Feedback Field-effect Transistors with Quasi-nonvolatile Memory States

Juhee Jeon, Kyoungah Cho* and Sangsig Kim*

Department of Electrical Engineering, Korea University, 145 Anam-ro, Seongbuk-gu, Seoul 02841, Republic of Korea

* Corresponding author. chochem@korea.ac.kr (K. C.); Tel.: +82-2-3290-3898 (K. C.); sangsig@korea.ac.kr (S. K.); Tel.: +82-2-3290-3245(S. K.)

S1. Charge carrier distributions in States 0 and 1

As shown in Fig. S1, electrons in the gated channel are depleted (accumulated) in State 0 (1). Thus, the electron concentration in the gated channel is higher by approximately 5×10^{18} cm^{-3} in State 0 than that in State 1. However, the hole concentrations in the nongated channel are the same in States 0 and 1.

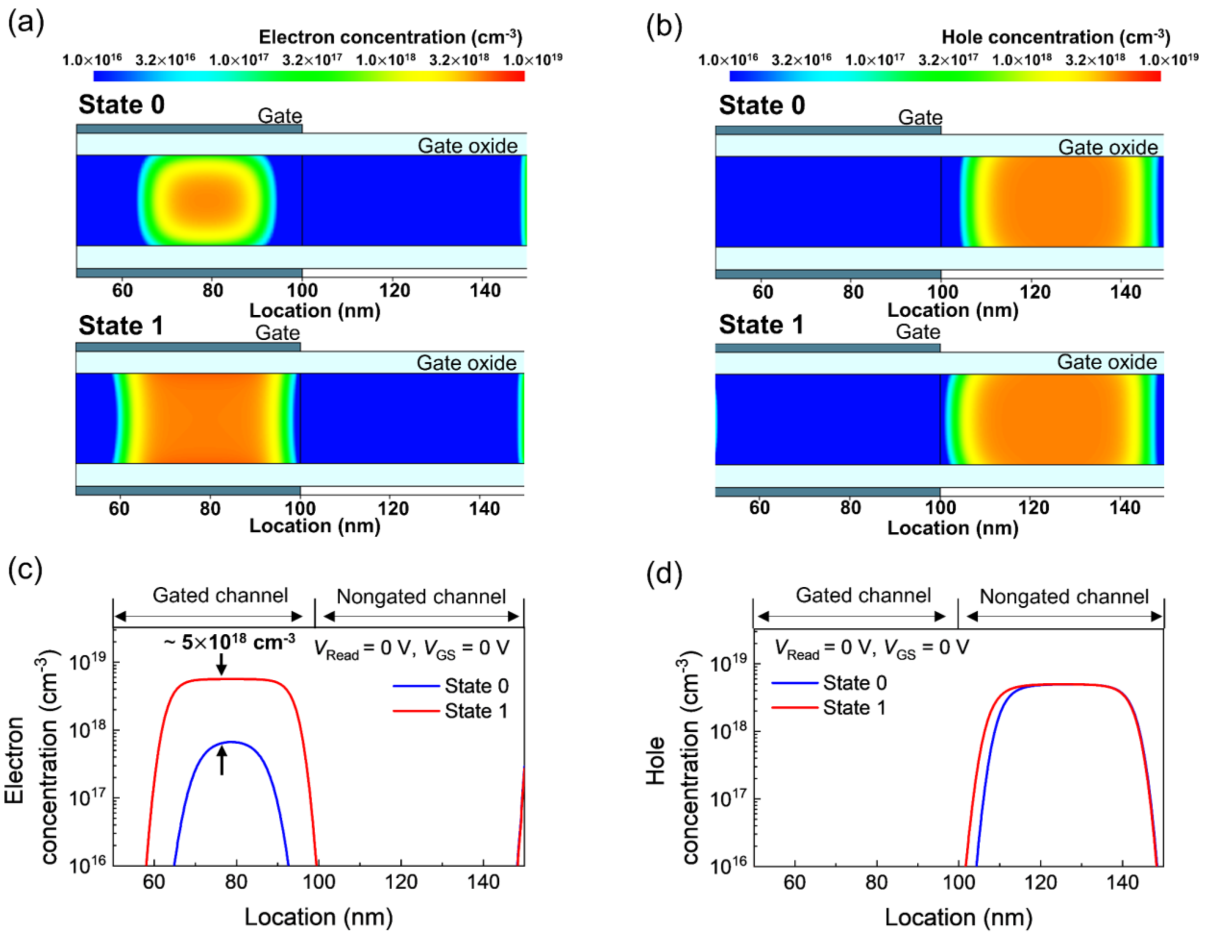

Figure S1. (a) Electron and (b) hole distributions in States 0 and 1. (c) Electron and (d) hole concentrations in States 0 and 1 at the 1 nm position below the gate oxide.

S2. Effect of read operation on charge carrier distributions in States 0 and 1

Figure S2 shows charge carrier distributions in States 0 and 1 before and after the read operation (the read pulse time = the 40 ns). In State 0, the electron and hole concentrations do not change substantially after the read operation. In State 1, the electron concentration increases significantly after the read operation, whereas the hole concentration remains the same before and after the read operation.

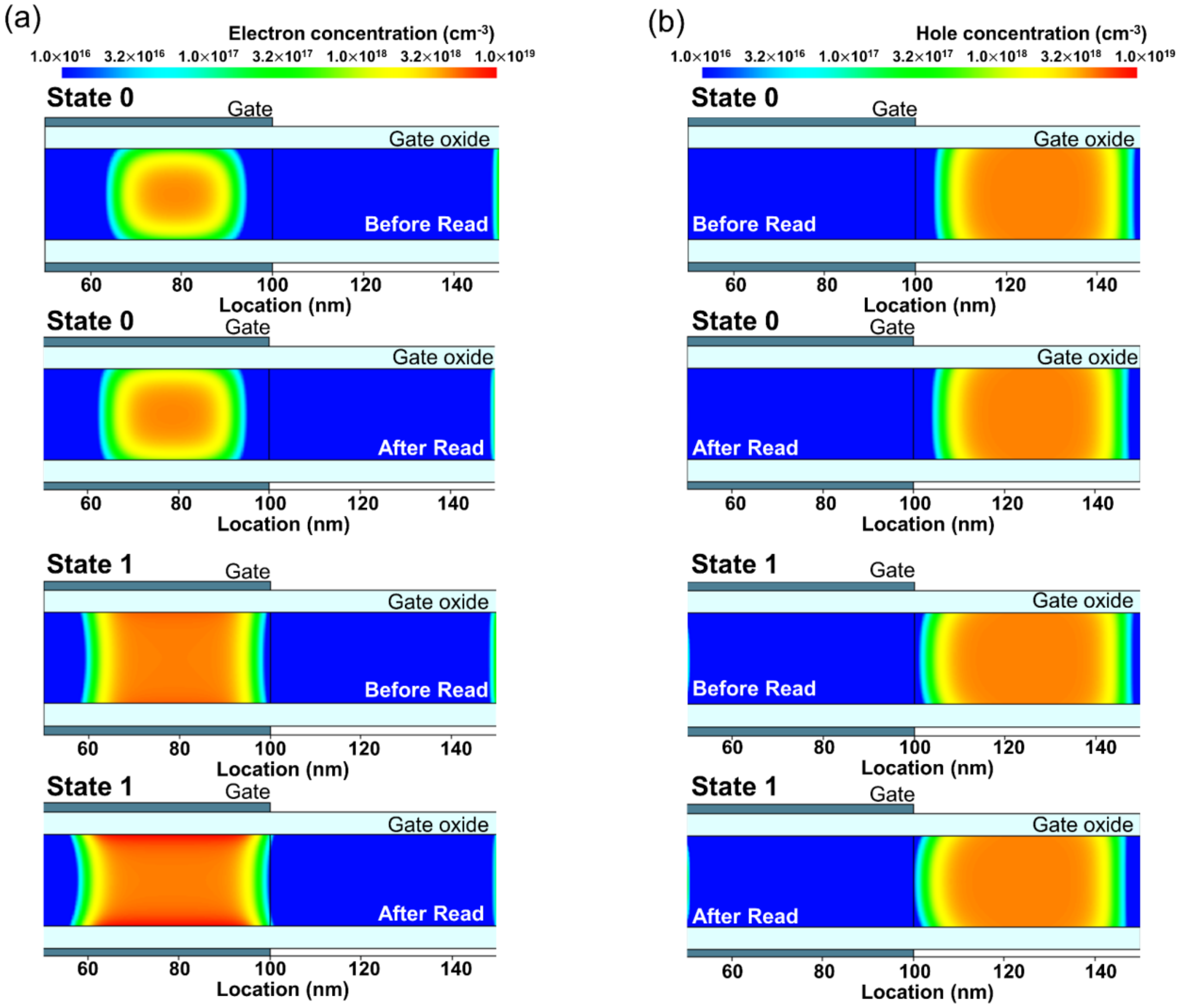

Figure S2. (a) Electron and (b) hole distributions in States 0 and 1 before and after the read operation.
